# Interpregnancy interval and perinatal outcomes across Latin America from 1990 to 2009: a large multi‐country study

**DOI:** 10.1111/1471-0528.13625

**Published:** 2015-09-24

**Authors:** LE Mignini, G Carroli, AP Betran, R Fescina, C Cuesta, L Campodonico, B De Mucio, KS Khan

**Affiliations:** ^1^ Centro Rosarino de Estudios Perinatales (CREP)RosarioArgentina; ^2^ UNDP/UNFPA/WHO/World Bank Special Programme of Research Development and Training in Human Reproduction (HRP)Department of Reproductive Health and ResearchWorld Health OrganizationGenevaSwitzerland; ^3^ Centro Latinoamericano de Perinatología (CLAP)MontevideoUruguay; ^4^ Women's Health Research UnitBarts and The London School of Medicine and DentistryQueen Mary University of LondonLondonUK

**Keywords:** Fetal death, interpregnancy intervals, maternal death, perinatal, pre‐eclampsia

## Abstract

**Objective:**

To determine the relationship of interpregnancy interval with maternal and offspring outcomes.

**Design:**

Retrospective study with data from the Perinatal Information System database of the Latin American Centre for Perinatology and Human Development, Uruguay.

**Setting:**

Latin America, 1990–2009.

**Population:**

A cohort of 894 476 women delivering singleton infants.

**Methods:**

During 1990–2009 the Perinatal Information System database of the Latin American Centre for Perinatology identified 894 476 women with defined interpregnancy intervals: i.e. the time elapsed between the date of the previous delivery and the first day of the last normal menstrual period for the index pregnancy. Using the interval 12–23 months as the reference category, multiple logistic regression estimated adjusted odds ratios (aORs) with 95% confidence intervals (95% CIs) of the association between various interval lengths and maternal and offspring outcomes.

**Main outcome measures:**

Maternal death, pre‐eclampsia, eclampsia, puerperal infection, fetal death, neonatal death, preterm birth, and low birthweight.

**Results:**

In the reference interval there was 0.05% maternal death, 1.00% postpartum haemorrhage, 2.80% pre‐eclampsia, 0.15% eclampsia, 0.28% puerperal infection, 3.45% fetal death, 0.68% neonatal death, 12.33% preterm birth, and 9.73% low birthweight. Longer intervals had increased odds of pre‐eclampsia (>72 months), fetal death (>108–119 months), and low birthweight (96–107 months). Short intervals of <12 months had increased odds of pre‐eclampsia (aOR 0.80; 95% CI 0.76–0.85), neonatal death (aOR 1.18; 95% CI 1.08–1.28), and preterm birth (aOR 1.16; 95% CI 1.11–1.21). Statistically, the interval had no relationship with maternal death, eclampsia, and puerperal infection.

**Conclusions:**

A short interpregnancy interval of <12 months is associated with pre‐eclampsia, neonatal mortality, and preterm birth, but not with other maternal or offspring outcomes. Longer intervals of >72 months are associated with pre‐eclampsia, fetal death, and low birthweight, but not with other maternal or offspring outcomes.

**Tweetable abstract:**

A short interpregnancy interval of <12 months is associated with neonatal mortality and preterm birth.

## Tweetable abstract

A short interpregnancy interval of <12 months is associated with neonatal mortality and preterm birth.

## Introduction

The last decades have seen the postponement of age at first birth, reduction in parity, and lengthening of birth intervals.^1^, ^2^ Birth spacing has shown fluctuations, including shifts towards shorter intervals.^3^ Generating public health guidance on birth spacing remains an important topic, but many studies that purport an association of short and long birth intervals with maternal and offspring outcomes are not from recent times.^4^, ^5^ Studies frequently ignore the inverse relationship that exists between maternal and offspring outcomes. If a mother is delivered early to prevent complications of pre‐eclampsia, the risk of maternal mortality may be lowered but that of offspring mortality is increased as a result of prematurity. Addressing this issue requires the simultaneous assessment of both outcomes in the same cohort, and the one study that did this had a small sample size of just 7897, risking imprecision and overfitting.^6^ These deficiencies threaten the validity of the findings in the literature and their current applicability, as reproductive behaviour and outcomes have changed considerably over time. Thus controversy remains about the factual information needed to underpin recommendations.^7^

We determined the relationship of interpregnancy interval (IPI) with maternal and offspring outcomes in the same cohort, applying multivariable analysis and adjusting for the effect of potential confounding factors in a large data set to generate reliable estimates of the association.

## Methods

We developed an analysis plan using recommended contemporaneous methods and followed existing guidelines for reporting.^8^

### Participants

We used a large, high‐quality, longitudinal, anonymised data set from the Perinatal Information System Database of the Latin American Centre for Perinatology and Women's Reproductive Health (CLAP/WR), Montevideo, Uruguay (www.clap.ops-oms.org/sistemas). Included in the database assembled for our study were parous women who delivered two consecutive infants over a 20‐year period between 1990 and 2009, from Argentina, Bahamas, Bolivia, Brazil, Chile, Colombia, Costa Rica, Dominican Republic, Ecuador, El Salvador, Honduras, Mexico, Nicaragua, Panama, Paraguay, Peru, Uruguay, and Venezuela. We excluded women with unknown dates of delivery and/or last menstrual periods. Moreover, pregnant women with IPIs of <3 months and >10 years were excluded in order to minimise the risk of data‐entry errors in the database.

### Definition of birth interval

The IPI was defined as the time elapsed between the date of the woman's previous delivery and the first day of the last normal menstrual period for the index pregnancy. We did not use the interbirth interval (time interval between the date of previous delivery and the birth date of the index pregnancy), as it may overestimate the risk of adverse offspring outcomes for very short intervals between pregnancies.^4^ The interval was calculated in days and converted into completed months (30.5 days was taken to equal 1 month). The IPIs were categorised as <12, 12–23, 24–35, 36–47, 48–59, 60–71, 72–83, 84–95, 96–107, and 108–119 months. The interval 12–23 months was set *a priori* as the reference category for statistical analysis, underpinned by our systematic review of the literature, where the most common reference interval was within the 12–24 months range, and this interval was likely to have the lowest rates of adverse outcomes.

### Outcome measures

Primary outcomes were maternal and offspring mortality. Maternal death was the death of a woman while she was pregnant or within 42 days after delivery from any cause related to or aggravated by the pregnancy or its management, but not from accidental or incidental causes. Offspring death included both fetal death (delivery of a dead baby at or after 20 weeks of gestation) and neonatal death (death of a liveborn infant within the first 28 days of life). Secondary maternal outcomes were the main causes of death, postpartum haemorrhage, pre‐eclampsia, eclampsia, and puerperal infection, classified in our database according to the International Classification of Diseases, 10th revision (ICD‐10).^5^ Secondary offspring outcomes were the main causes of neonatal death,^9^ low birthweight (live baby weighing less than 2.5 kg at birth), and preterm birth (live baby delivered before 37 weeks of gestation, defined as the time between the date of the mother's last menstrual period and the infant's birth date).

### Statistical analyses

Rates of maternal and offspring outcomes were calculated for each IPI. For computing measures of association of the outcomes in various IPIs versus the outcomes in the 12–23 months reference interval, the influence of known and suspected measured confounding factors was controlled for multivariable logistic regression modelling in order to derive adjusted odds ratios (aORs) with 95% confidence intervals (95% CI).^10^, ^11^ Models were built for each outcome separately, incorporating a range of independent variables appropriate for the adjustment of the association between IPI and that outcome. The selection process for variables was driven by causal knowledge for the adjustment of confounding.^12^ We used forward stepwise regression, with maternal age forced in to the model. The variables for maternal and offspring models included maternal age, previous pre‐eclampsia, previous eclampsia, previous caesarean section, previous early neonatal mortality, parity, diabetes, urinary infection, hypertension during first, second, and third trimester, and singleton birth. A complete list of the final set of covariates is provided with each model in the results section. The modelling was conducted both with the imputation of missing values and after excluding cases with missing data (results for the latter analysis are provided in Appendix S1, for comparison).^13^ The multiple imputation methods were used.^14^ We first created a monotone missing pattern using a Markov chain Monte Carlo (MCMC) method, which assumes multivariate normality, to impute all missing values or just enough missing values to make the imputed data sets have monotone missing patterns. The second step used more specific techniques (for the imputed data set with a monotone missing pattern), depending on the variable. For continuous variables we checked for normality and then we used a linear regression. We used logistic regression for categorical variables, and discriminant analysis for nominal variables. Nineteen variables (outcomes and predictors) were imputed.^13^ Nine variables had less than 10% of missing values, whereas the remaining variables had between 10 and 15% of missing values. The sas procedure proc mi was used to create 25 complete data sets with imputed values to fill in the missing values.^14^ A logistic model was selected (for each outcome separately) using a forward stepwise method in each imputed data set (the significance level in the model was set at 0.05). Variables selected in at least 20 imputed data sets were retained for inclusion in the final model. With the 25 completed data sets, the sas procedure proc mianalyze was used in conjunction with sas procedure proc logistic to adjust the final models. Modelling for the secondary outcome, postpartum haemorrhage, is not reported because of the large proportion of missing data (72%). Analyses were performed with the sas statistical package (version 9.3; SAS Institute Inc., Cary, NC, USA). Statistical tests were two‐sided, and *P* < 0.05 was used to indicate statistical significance.

## Results

**Figure 1:**
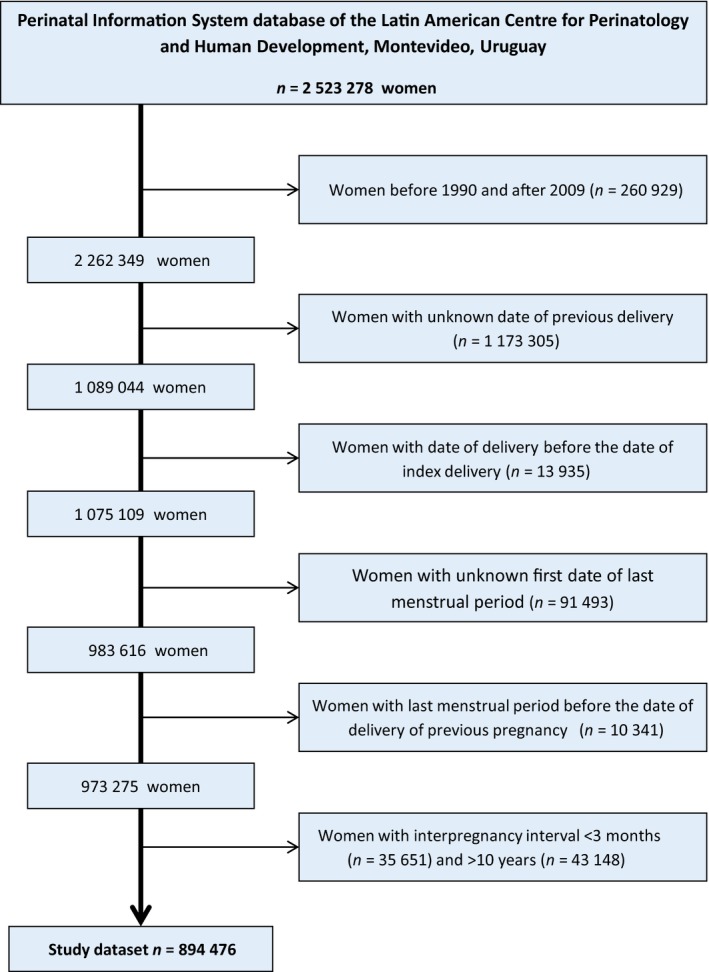
Flowchart of construction of database for the study of the relationship of interpregnancy interval with maternal and perinatal outcomes in a cohort of women delivering two consecutive infants in the period 1990–2009.

The data set included 894 476 women whose records contained complete information to calculate IPI (Figure 1). The distribution of the interval was skewed, with the median interval at 28 months (interquartile range 15–51 months), and peaking within the 12–23 month interval consistently in each of the four 5‐year time periods covered (Figure S1). Short (<12 months), reference (12–23 months), intermediate (24–59 months), and long (≥60 months) intervals between pregnancies were observed for 17.4, 25.6, 37.9, and 19.1% of women, respectively (Table S1).

**Figure 2:**
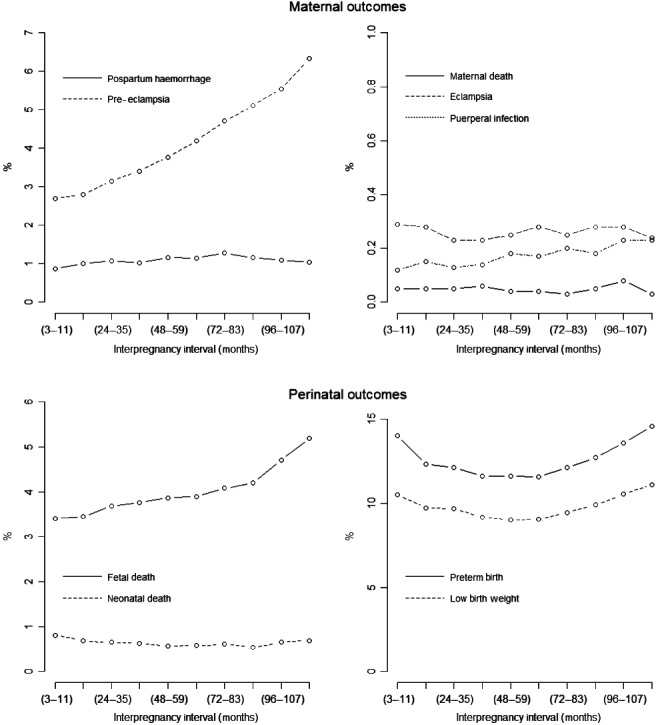
Rates of maternal and perinatal outcomes according to interpregnancy interval in a cohort of 894 476 women delivering two consecutive infants in the period 1990–2009.

The baseline characteristics of the mothers at the index pregnancy varied according to the IPI (Table S1), as did the rates of the outcomes (Figure 2; Tables S2 and S3). Among index pregnancies in the 12–23 month reference interval there was 0.05% maternal death (50 maternal deaths per 100 000 live births), 1.00% postpartum haemorrhage, 2.80% pre‐eclampsia, 0.15% eclampsia, 0.28% puerperal infection, 3.45% fetal death, 0.68% neonatal death, 12.33% preterm birth, and 9.73% low birthweight (Table S2). On a graphical examination of crude rates, pre‐eclampsia and fetal death appeared to increase linearly, whereas low birthweight and preterm birth appeared to have a shallow U‐shaped distribution (Figure 2).

The statistical assessment of the association showed no relationship between the interval and maternal or fetal death (Tables 1 and 2). It showed that compared with mothers with IPIs of 12–23 months, mothers with intervals <12 months and >72 months had increased odds of pre‐eclampsia. The odds ratios for pre‐eclampsia increased as the interval became longer (e.g. aOR 1.1, 95% CI 1.02–1.18 at 72–83 months; aOR 1.15, 95% CI 1.06–1.24 at 84–95 months; aOR 1.18, 95% CI 1.09–1.27 at 96–107 months). There was no significant association between the interval and eclampsia and puerperal infection. With 72% missing outcome data, the findings of modelling for the secondary outcome postpartum haemorrhage were not reported. Regarding offspring outcomes, compared with mothers with IPIs of 12–23 months, women with short intervals had increased odds of neonatal death (aOR 1.18; 95% CI 1.08–1.28) and preterm birth (aOR 1.16; 95% CI 1.11–1.21). Although there was no significant association with shorter IPIs, longer intervals had increased odds of fetal death (>108–119 months) and low birthweight (96–107 months).

**Table 1:** Adjusted odds ratio (95% confidence interval) for different maternal outcomes according to interpregnancy interval in a cohort of 894 476 women delivering two consecutive infants 1990–2009

Interval (months)	Maternal death^a^	Preeclampsia^b^	Eclampsia^c^	Puerperal infection^d^
3–11	1.19 (0.88–1.60)	0.80 (0.76–0.85)	0.83 (0.63–1.08)	1.05 (0.87–1.26)
12–23	Ref	Ref	Ref	Ref
24–35	1.12 (0.88–1.43)	0.88 (0.84–0.92)	0.93 (0.81–1.08)	0.94 (0.65–1.36)
36–47	1.19 (0.82–1.75)	0.91 (0.86–0.97)	0.94 (0.69–1.28)	0.96 (0.68–1.35)
48–59	0.91 (0.65–1.27)	0.97 (0.92–1.02)	1.02 (0.79–1.31)	0.94 (0.65–1.35)
60–71	0.96 (0.70–1.32)	1.04 (0.98–1.09)	1.04 (0.83–1.31)	1.10 (0.92–1.31)
72–83	0.68 (0.36–1.27)	1.10 (1.02–1.18)	1.12 (0.86–1.46)	0.93 (0.58–1.51)
84–95	1.04 (0.66–1.64)	1.15 (1.06–1.24)	1.03 (0.74–1.43)	1.14 (0.79–1.64)
96–107	1.29 (0.68–2.46)	1.18 (1.09–1.27)	1.11 (0.81–1.51)	1.02 (0.65–1.60)
108–119	0.71 (0.32–1.55)	1.30 (1.20–1.41)	1.08 (0.73–1.60)	0.93 (0.59–1.46)

Multivariable logistic regression used for each outcome as dependent variable and various interpregnancy intervals as independent variables, using the interval 12–23 months as reference and applying multiple imputation for missing values (see Methods for details).

a Covariates in final maternal death model: Maternal age (continuous variable), parity (categorized as 0, 1–3, ≥4), singleton birth, eclampsia, hemorrhage in 3rd trimester.

b Covariates in final preeclampsia model: Maternal age (continuous variable), parity (categorized as above), singleton birth, diabetes, urinary infection, and previous early neonatal mortality, hemorrhage in 1st, 2nd, 3rd trimesters, and previous caesarean.

c Covariates in final eclampsia model: Maternal age (continuous variable), singleton birth, diabetes, urinary infection, and previous early neonatal mortality, hemorrhage in 1st, 2nd, 3rd trimesters, and previous caesarean.

d Covariates in final puerperal infection model: Maternal age (continuous variable), parity (categorized as above), singleton birth, preeclampsia, eclampsia, urinary infection, hemorrhage in 1st, 2nd, 3rd trimester and previous early neonatal mortality, and previous caesarean.

**Table 2:** Adjusted odd ratios (95% confidence interval) for different perinatal outcomes according to interpregnancy interval in a cohort of 894 476 women delivering two consecutive infants 1990–2009

Interval (months)	Fetal death	Neonatal death	Low birth weight	Preterm birth
3–11	1.00 (0.93–1.07)	1.18 (1.08–1.28)	1.03 (0.94–1.14)	1.16 (1.11–1.21)
12–23	Ref	Ref	Ref	Ref
24–35	0.99 (0.94–1.03)	1.00 (0.93–1.08)	0.99 (0.95–1.03)	0.97 (0.94–1.00)
36–47	0.99 (0.95–1.03)	0.99 (0.92–1.06)	0.96 (0.90–1.02)	0.93 (0.90–0.95)
48–59	0.99 (0.95–1.04)	0.93 (0.86–1.01)	0.93 (0.89–0.98)	0.92 (0.90–0.94)
60–71	0.94 (0.89–0.99)	0.96 (0.87–1.05)	0.94 (0.90–0.98)	0.91 (0.88–0.94)
72–83	0.97 (0.91–1.03)	0.97 (0.87–1.08)	0.98 (0.93–1.01)	0.95 (0.92–0.98)
84–95	0.97 (0.90–1.05)	0.90 (0.79–1.02)	1.03 (1.00–1.07)	1.00 (0.97–1.04)
96–107	1.04 (0.96–1.13)	1.01 (0.88–1.17)	1.07 (1.01–1.13)	1.06 (1.00–1.11)
108–119	1.14 (1.03–1.27)	1.07 (0.91–1.24)	1.12 (1.04–1.19)	1.12 (1.05–1.19)

Multivariable logistic regression used for each outcome as dependent variable and various interpregnancy intervals as independent variables, using the interval 12–23 months as reference and applying multiple imputation for missing values (see Methods for details). Covariates in all final models: Maternal age (continuous variable), parity (categorized as 0, 1–3, ≥4), singleton birth, diabetes, preeclampsia, eclampsia, urinary infection, hemorrhage at 1st, 2nd and 3rd trimesters, and previous early neonatal mortality and previous caesarean.

The findings of multivariable logistic regression using complete case analyses (without imputation for missing values) were consistent with the results above, except that in the relationship of long intervals with pre‐eclampsia the odds increased after >24 months, and the short intervals of <12 months had increased odds of low birthweight (aOR 1.07; 95% CI 1.04–1.10) (Appendix S1).

## Discussion

### Main findings

Our results indicate that short IPIs of <12 months were associated with neonatal mortality and preterm birth, but not with maternal outcomes. Longer intervals of >72 months were associated with pre‐eclampsia, but not with other maternal or offspring outcomes. Birth spacing covers a reproductive continuum, including conception, pregnancy, birth, breastfeeding, and family planning. The risk of neonatal mortality and preterm birth linked to short intervals is small, but this finding is important because preterm birth is predicted to become the leading proportional cause of child deaths.^9^, ^15^ The rising rates with length of IPI of pre‐eclampsia, the second most common cause of maternal mortality worldwide,^16^ underscores the importance of the persistence needed for improving coverage of obstetric care. The finding that maternal mortality is not linked with the length of IPI emphasises the role of family planning and breastfeeding in promoting safe motherhood and achieving better offspring outcomes.^17^, ^18^

### Review of the literature

We undertook a review to determine the relationship of birth interval with outcomes in both mother and baby. Citations were identified without language restriction through the electronic databases MEDLINE, EMBASE, and LILACS (from database inception to March 2011), bibliographies of retrieved articles and known reviews, and contact with experts. From 2364 initial citations, 117 articles met the selection criteria. Studies were from 57 countries between 1958 and 2010. They were heterogeneous in: settings (developed 26.5%; developing 70.9%; mixed 2.6%); definitions of birth interval (interpregnancy 53%; interbirth 47%); specification of reference interval for comparison (mode 12–24 months; range <3 months and >36 months); outcome and outcome measurements (three maternal and eight baby outcomes); design (cohort 57.5%; cross‐sectional 23.8%; case–control 18.7%); and methodological quality (high 50.4%; low 49.6%). No subgroup was large enough to benefit from the precision gained by meta‐analysis. Based on vote counting, it was possible to observe that shorter intervals (<12–18 months) were associated with maternal mortality, miscarriage, low birthweight, preterm birth, and offspring mortality, whereas longer intervals (>24 months) were associated with maternal mortality, pre‐eclampsia, miscarriage, preterm birth, and offspring mortality. Short and long birth spacing intervals appeared to be associated with poorer outcomes, both for mothers and babies, but deficiencies arising from heterogeneity and bias left considerable uncertainty about the trustworthiness of these findings. Thus the existing literature appeared weak for making specific recommendations regarding optimal birth spacing and for setting thresholds at the ends of a safe interval. Our study addressed the existing deficiencies by addressing the relationship of pre‐defined intervals with core maternal and offspring outcomes in the same cohort. It did not demonstrate a U‐shaped association. It showed that short IPIs of <12 months were associated with neonatal mortality and preterm birth, but not with maternal outcomes. Long intervals of >72 months were associated with pre‐eclampsia, but not with other maternal or offspring outcomes.

### Strengths and limitations

Our findings are supported by an *a priori* analysis plan, with pre‐specified outcome variables and reference IPI. The large sample size and the use of multiple imputations for handling missing data confer reasonable statistical power to reliably evaluate the relationship of interest. Even for outcomes with low event rates, like maternal mortality, we had enough data to meet the 10 events per variable rule to avoid over‐fitting the models.^10^, ^11^ The use of multiple imputations for handling missing data, and the control that we were able to exert on the influence of many potential confounding factors, adds strength to the validity of our observations; however, as many factors are unknown, unmeasured, or poorly measured, the adjustment for confounding had some deficiencies. For example, long intervals associated with adverse maternal outcomes may be linked to poorer health at older maternal ages, but we did not have data on maternal weight, body mass index (BMI), and hypertension for inclusion in the models; we did have data on maternal age and diabetes to include in the models. The proportion of cases missing was particularly large for some variables, e.g. nearly two‐thirds were missing for postpartum haemorrhage and country, and so we could not model with these variables. One way to minimise the data excluded for missing information on last menstrual period could have involved estimating IPI in a different way (i.e. by date of the second birth minus date of the first birth minus gestational age in weeks of the second birth); we were unable to implement this as a sensitivity analysis because of limited resources. Regarding the statistical handling of maternal age, higher‐order polynomials or splines could have been used to ensure that the observed associations were not capturing components of some association linked with advanced maternal age; we were unable to implement this because of limited resources. Another concern arises from the need to maintain anonymity, which meant that we could not employ unique identifiers in the analysis. In the absence of relevant tracking data other than identifiers we were forced to assume that pregnancy pairs were independent. As a result of the observational design of our study and the limitations mentioned above, a causal association may not be inferred, but it merits consideration, particularly as mechanisms exist to explain the link between short intervals and poor outcomes.^12^ Yet another limitation of our study was that many women have no known last menstrual period and the estimated date of delivery was assigned based on ultrasound. This may introduce error in the estimated gestational age analysed, as ultrasound may be more accurate than last menstrual period‐based dating in many cases. Our data are from multiple countries gathered over a relatively recent time period with birth intervals more realistic for the current time, for example around 60% of the women had an IPI of <3 years. This adds to the generalisability of our findings.

### Interpretation

Some of our results corroborate the findings from earlier reports, whereas others challenge the prevailing wisdom. We were unable to replicate the deep U‐shaped association previously seen for IPI and maternal and offspring outcomes.^4^, ^5^ It may be speculated that data exploration can optimise the definitions of birth intervals, their cut‐offs and groupings for comparisons, the choice of reporting of outcomes, and the selection of variables for the control of confounding to maximise the likelihood of reaching statistically significant results that fit the U‐shaped association hypothesis. We do not make this point to criticise previous research. We simply want to emphasise the importance of an *a priori* analysis plan in observational studies.^8^ We focused on pre‐specified core and important outcomes for both mother and baby simultaneously using predefined intervals. For pre‐eclampsia, we found an increase in odds as intervals got longer. This trend cannot simply be explained by the association of maternal characteristics (such as age) with IPI, as controlling for these factors was incorporated into our model. Women with short, but not long, IPIs had increased odds of neonatal death and preterm birth. Many causal mechanisms exist for these associations.^12^ One hypothesis is that maternal nutritional depletion through close succession of pregnancies and lactations arising because of insufficient time for replenishment may increase the risk of adverse offspring outcomes.^19^ For example, the lack of replenishment of the physiological depletion of folate that occurs in pregnancy and lactation may lead future pregnancies to be conceived under a state of folate deficiency, thereby increasing the risks of neural tube defects, fetal growth restriction, and preterm birth.^20^

## Conclusion

In conclusion, women seeking advice on birth spacing in Latin America can be reassured that short intervals of <12 months and longer intervals of >24 months are both generally safe for the mother, except for the odds of pre‐eclampsia, which increase as the interval increases in length. They can be warned that short IPIs of <12 months are associated with a small risk of neonatal mortality and morbidity, but that longer intervals >24 months are safe for the baby. These data provide reliable information to underpin discussions about the spacing of pregnancies.

### Disclosure of interests

Full disclosure of interests available to view online as supporting information.

### Contribution to authorship

The study was conceived and designed by all co‐authors. CC performed the statistical analysis. LM and CC had full access to all data in the study and take responsibility for the integrity of the data and accuracy of the data analysis. They also affirm that the article is an honest, accurate, and transparent account of the study being reported, that no important aspects of the study have been omitted, and that any discrepancies from the study as planned have been explained. All authors (LEM, GC, APB, RF, CC, LC, BDM, and KSK) commented on the article.

### Details of ethics approval

This study received approval from the CREP Data Protection Board and the Scientific Ethics Committees.

### Funding

Financial disclosures: none to report. Funding/support: this study was funded by the UNDP/UNFPA/WHO/World Bank Special Programme of Research, Development and Research Training in Human Reproduction, Department of Reproductive Health and Research, World Health Organization. KSK and LM used funding from the European Union made available to the Evidence‐Based Medicine COllaboratioN: NEtwork for systematic reviews and guideline development researCh and dissemination (EBM‐CONNECT) Collaboration through its Seventh Framework Programme, Marie Curie Actions, International Staff Exchange Scheme (proposal no. 101377; grant agreement no. 247613) for data interpretation and drafting the article. The sponsor was the UNDP/UNFPA/WHO/World Bank Special Program of Research, Development and Research Training in Human Reproduction, Department of Reproductive Health and Research, World Health Organization. The study was designed, conducted, analysed, and interpreted by the authors, independent of funding sources and the sponsor. Neither the funding sources nor the sponsor played a role in: the design and conduct of the study; the collection, management, analysis, and interpretation of the data; or the preparation, review, and approval of the article.

## Supporting information


**Figure S1.** Distribution of interpregnancy intervals (in months) in a cohort of 894 476 women delivering two consecutive infants by 5‐year time periods during the period 1990–2009.

**Table S1.** Distribution of sociodemographic and obstetric characteristics according to interpregnancy interval in a cohort of 894 476 women delivering two consecutive infants during the period 1990–2009.
**Table S2.** Rates of maternal outcomes according to interpregnancy interval in a cohort of 894 476 women delivering two consecutive infants in the period 1990–2009.
**Table S3.** Rates of adverse perinatal outcomes according to interpregnancy interval in a cohort of 894 476 women delivering two consecutive infants in the period 1990–2009.

**Appendix S1.** Multivariate logistic regression with complete case analysis (without multiple imputation for missing values).
