# Supplementary material for: Interpregnancy interval and perinatal outcomes across Latin America from 1990 to 2009: a large multi‐country study
Source: BJOG. 2015 Sep 24;123(5):730–7. doi: 10.1111/1471-0528.13625 (PMC4949506; doi:10.1111/1471-0528.13625)
Supplement: Supplementary file 1 — Figure S1. Distribution of interpregnancy intervals (in months) in a cohort of 894 476 women delivering two consecutive infants by 5‐year time periods during the period 1990–2009. [file BJO-123-730-s001.pdf]

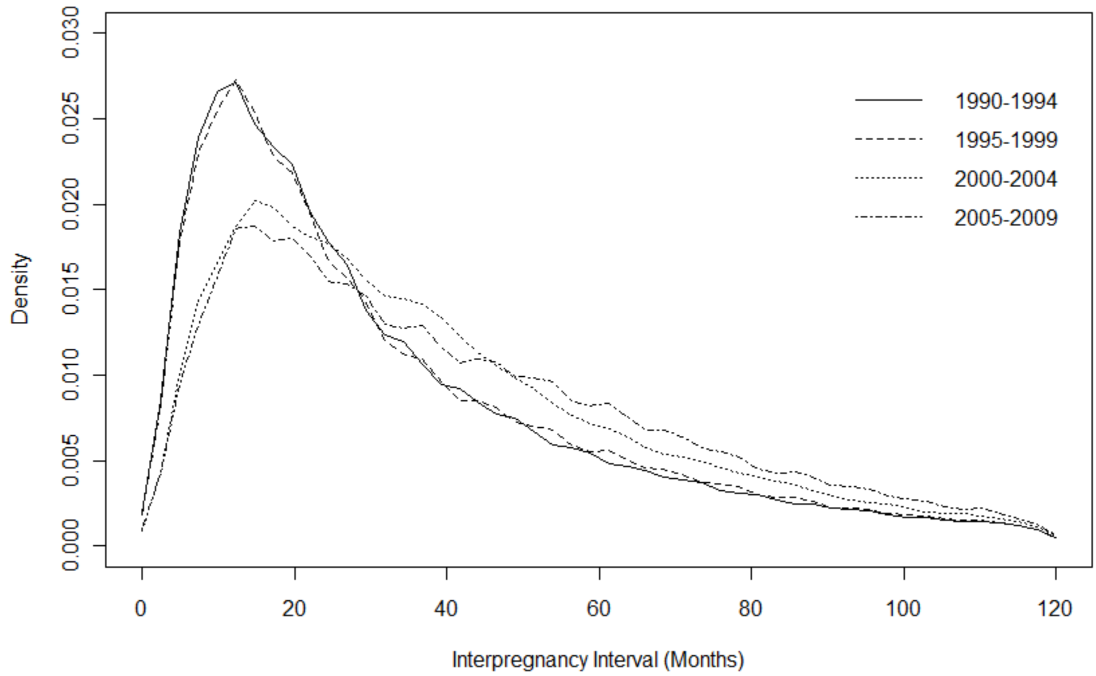


**Figure S1.** Distribution of interpregnancy intervals (in months) in a cohort of 894,476 women delivering two consecutive infants by 5-year time periods 1990-2009.
