# Supplementary material for: Interpregnancy interval and perinatal outcomes across Latin America from 1990 to 2009: a large multi‐country study
Source: BJOG. 2015 Sep 24;123(5):730–7. doi: 10.1111/1471-0528.13625 (PMC4949506; doi:10.1111/1471-0528.13625)
Supplement: Supplementary file 2 — Table S1. Distribution of sociodemographic and obstetric characteristics according to interpregnancy interval in a cohort of 894 476 women delivering two consecutive infants during the period 1990–2009. Table S2. Rates of maternal outcomes according to interpregnancy interval in a cohort of 894 476 women delivering two consecutive infants in the period 1990–2009. Table S3. Rates of adverse perinatal outcomes according to interpregnancy interval in a cohort of 894 476 women delivering two consecutive infants in the period 1990–2009. [file BJO-123-730-s002.pdf]

Table S1. Distribution of sociodemographic and obstetric characteristics according to interpregnancy interval in a cohort of 894,476 women delivering two consecutive infants 1990-2009

	Interpregnancy interval (months)									
	3-11	12-23	24-35	36-47	48-59	60-71	72-83	84-95	96-107	108-119
N (%)	155958 (17.4)	228455 (25.6)	151240 (16.9)	107392 (12.0)	80090 (9.0)	59188 (6.6)	43098 (4.8)	30698 (3.4)	22034 (2.5)	16323 (1.8)
Maternal age (years)										
<17	3129 (2.0)	2086 (0.9)	506 (0.3)	164 (0.2)	67 (0.1)	51 (0.1)	21 (0.1)	14 (0.1)	9 (0.0)	5 (0.0)
17-19	22260 (14.3)	25000 (10.9)	10395 (6.9)	3745 (3.5)	1094 (1.4)	360 (0.6)	143 (0.3)	72 (0.2)	53 (0.2)	28 (0.2)
20-24	54266 (34.8)	77955 (34.1)	50266 (33.2)	33109 (30.8)	20304 (25.4)	11035 (18.6)	5371 (12.5)	2169 (7.1)	796 (3.6)	365 (2.2)
25-29	38285 (24.6)	59779 (26.2)	43163 (28.6)	33892 (31.6)	27920 (34.9)	21293 (36.0)	15369 (35.7)	10066 (32.8)	6314 (28.7)	3723 (22.8)
30-34	23356 (15.0)	37765 (16.5)	27439 (18.1)	21917 (20.4)	18432 (23.0)	15685 (26.5)	12733 (29.5)	10365 (33.8)	8099 (36.8)	6301 (38.6)
>34	13945 (8.9)	24666 (10.8)	18643 (12.3)	13938 (13.0)	11766 (14.7)	10377 (17.53)	9184 (21.3)	7809 (25.4)	6601 (30.0)	5776 (35.4)
Missing data	717 (0.5)	1204 (0.5)	828 (0.6)	627 (0.6)	507 (0.6)	387 (0.7)	277 (0.6)	203 (0.7)	162 (0.7)	125 (0.8)
No of previous deliveries										
0	7896 (5.06)	15554 (6.8)	13246 (8.7)	11457 (10.7)	9585 (11.9)	7532 (12.7)	5334 (12.4)	3536 (11.5)	2342 (10.6)	1605 (9.8)
1-3	109556 (70.2)	155447 (68.0)	104075 (68.8)	74720 (69.6)	55574 (69.4)	40703 (68.8)	29610 (68.7)	21295 (69.4)	15366 (69.7)	11449 (70.4)
>4	38382 (24.6)	57306 (25.0)	33835 (22.4)	21155 (19.7)	14907 (18.6)	10935 (18.5)	8139 (18.9)	5855 (19.0)	4320 (19.6)	3260 (20.0)
Missing data	124 (0.08)	148 (0.06)	84 (0.06)	60 (0.06)	24 (0.03)	18 (0.03)	15 (0.03)	12 (0.04)	6 (0.03)	9 (0.06)
History of miscarriage										
Yes	39482 (25.3)	49640 (21.7)	32583 (21.5)	22934 (21.4)	17219 (21.5)	12890 (21.8)	9853 (22.9)	7251 (23.6)	5307 (24.0)	4205 (25.8)
No	113218 (72.6)	173442 (75.9)	115002 (76.0)	81823 (76.2)	60938 (76.0)	44819 (75.7)	32222 (74.8)	22661 (73.8)	16161 (73.3)	11722 (71.8)
Missing data	3258 (2.09)	5373 (2.35)	3655 (2.42)	2635 (2.45)	1933 (2.41)	1479 (2.50)	1023 (2.37)	786 (2.56)	566 (2.57)	396 (2.43)
History of fetal death										
Yes	10835 (6.95)	12763 (5.6)	7331 (4.8)	4496 (4.2)	3174 (3.9)	2137 (3.6)	1543 (3.6)	1094 (3.6)	808 (3.7)	576 (3.5)
No	136918 (87.8)	203305 (89)	135533 (89.6)	96708 (90.0)	72341 (90.3)	53566 (90.5)	38988 (90.5)	27690 (90.2)	19854 (90.1)	14720 (90.2)
Unknown	8205 (5.3)	12387 (5.4)	8376 (5.5)	6188 (5.8)	4575 (5.7)	3485 (5.9)	2567 (5.9)	1914 (6.2)	1372 (6.2)	1027 (6.3)

Mother's education										
None	6064	12315	8257	5707	4352	3004	2161	1527	949	664
	(3.9)	(5.4)	(5.5)	(5.3)	(5.4)	(5.1)	(5.0)	(5.0)	(4.3)	(4.1)
Primary	77664	114549	72880	50232	37046	27265	19791	13985	10072	7312
	(49.8)	(50.1)	(48.2)	(46.8)	(46.3)	(46.1)	(45.9)	(45.6)	(45.7)	(44.8)
Secondary	59622	83039	56832	41927	31479	23657	17322	12445	9020	6782
	(38.2)	(36.3)	(37.6)	(39.0)	(39.3)	(40.0)	(40.2)	(40.5)	(41.0)	(41.5)
University	7367	10942	8023	5719	4421	3209	2311	1670	1193	902
	(4.7)	(4.8)	(5.3)	(5.3)	(5.5)	(5.4)	(5.4)	(5.4)	(5.4)	(5.5)
Unknown	5241	7610	5248	3807	2792	2053	1513	1071	800	663
	(3.4)	(3.3)	(3.5)	(3.5)	(3.5)	(3.5)	(3.5)	(3.5)	(3.6)	(4.1)

Table S2. Rates of maternal outcomes according to interpregnancy interval in a cohort of 894,476 women delivering two consecutive infants 1990-2009.

Interval (months)	Maternal Death	Postpartum Haemorrhage	Preeclampsia	Eclampsia	Puerperal Infection
3-11	69/144694 (0.05%)	258/29673 (0.87%)	3573/132442 (2.70%)	158/132105 (0.12%)	376/130725 (0.29%)
12-23	101/213744 (0.05%)	571/57000 (1.00%)	5471/195685 (2.80%)	288/195153 (0.15%)	540/192717 (0.28%)
24-35	70/142393 (0.05%)	489/45529 (1.07%)	4082/129645 (3.15%)	172/129273 (0.13%)	290/127645 (0.23%)
36-47	58/101641 (0.06%)	381/36852 (1.03%)	3147/92713 (3.41%)	124/91829 (0.14%)	212/90653 (0.23%)
48-59	29/76014 (0.04%)	343/29573 (1.16%)	2589/68814 (3.76%)	121/68521 (0.18%)	170/67597 (0.25%)
60-71	25/56182 (0.04%)	254/22205 (1.14%)	2114/50294 (4.20%)	84/50069 (0.17%)	140/49384 (0.28%)
72-83	11/40919 (0.03%)	207/16219 (1.28%)	1731/36728 (4.71%)	73/36525 (0.20%)	91/36023 (0.25%)
84-95	16/29116 (0.05%)	133/11483 (1.16%)	1326/25947 (5.11%)	47/25816 (0.18%)	71/25389 (0.28%)
96-107	17/20874 (0.08%)	87/7961 (1.09%)	1030/18593 (5.54%)	42/18508 (0.23%)	51/18205 (0.28%)
108-119	5/15409 (0.03%)	59/5695 (1.04%)	876/13831 (6.33%)	31/13737 (0.23%)	32/13518 (0.24%)

Table S3. Rates of adverse perinatal outcomes according to interpregnancy interval in a cohort of 894,476 women delivering two consecutive infants 1990-2009.

Interval (months)	Fetal Death	Neonatal Death	Preterm (<37 weeks)	Low Birth Weight (<2500 g)
3-11	5251/153802 (3.41%)	1055/130964 (0.81%)	20612/148011 (14.00%)	15685/149261 (10.51%)
12-23	7761/225282 (3.45%)	1307/193402 (0.68%)	26719/216721 (12.33%)	21144/217266 (9.73%)
24-35	5504/148982 (3.69%)	826/127900 (0.65%)	17376/143634 (12.10%)	13796/142595 (9.67%)
36-47	3975/105685 (3.76%)	574/91271 (0.63%)	11874/102180 (11.62%)	9242/100946 (9.16%)
48-59	3047/78788 (3.87%)	384/68118 (0.56%)	8852/76270 (11.61%)	6783/75477 (8.99%)
60-71	2263/58231 (3.89%)	290/50228 (0.58%)	6516/56314 (11.57%)	5042/55817 (9.03%)
72-83	1728/42392 (4.08%)	224/36508 (0.61%)	4978/41056 (12.12%)	3832/40594 (9.44%)
84-95	1266/30127 (4.20%)	137/25875 (0.53%)	3696/29122 (12.69%)	2862/28894 (9.91%)
96-107	1016/21626 (4.70%)	121/18387 (0.66%)	2844/20936 (13.58%)	2185/20763 (10.52%)
108-119	830/15968 (5.20%)	93/13418 (0.69%)	2251/15484 (14.54%)	1698/15313 (11.09%)
