# Supplementary material for: Interpregnancy interval and perinatal outcomes across Latin America from 1990 to 2009: a large multi‐country study
Source: BJOG. 2015 Sep 24;123(5):730–7. doi: 10.1111/1471-0528.13625 (PMC4949506; doi:10.1111/1471-0528.13625)
Supplement: Supplementary file 3 — Appendix S1. Multivariate logistic regression with complete case analysis (without multiple imputation for missing values). [file BJO-123-730-s003.pdf]

Appendix S1. Multivariate logistic regression with complete case analysis (without multiple imputation for missing values).

Table 1A

Adjusted odds ratio (95% confidence interval) for different maternal outcomes according to interpregnancy interval in a cohort of 894,476 women delivering two consecutive infants 1990-2009.

Interval (months)	Maternal Death[a]	Preeclampsia[b]	Eclampsia[c]	Puerperal Infection[d]
3-11	1.24 (0.87-1.76)	0.99 (0.95-1.04)	0.81 (0.65-1.00)	0.98 (0.85-1.13)
12-23	Ref	Ref	Ref	Ref
24-35	1.16 (0.81-1.65)	1.08 (1.03-1.13)	0.93 (0.75-1.15)	0.79 (0.68-0.92)
36-47	1.20 (0.81-1.78)	1.14 (1.08-1.19)	0.89 (0.70-1.12)	0.84 (0.71-0.99)
48-59	0.81 (0.50-1.33)	1.18 (1.12-1.25)	1.08 (0.84-1.37)	0.91 (0.76-1.09)
60-71	0.99 (0.59-1.65)	1.25 (1.18-1.32)	0.96 (0.73-1.28)	1.06 (0.87-1.29)
72-83	0.67 (0.34-1.30)	1.32 (1.24-1.40)	1.24 (0.93-1.66)	0.94 (0.74-1.19)
84-95	1.10 (0.59-2.06)	1.40 (1.31-1.50)	1.12 (0.80-1.58)	1.10 (0.85-1.42)
96-107	1.35 (0.71-2.58)	1.44 (1.34-1.55)	1.26 (0.87-1.82)	0.96 (0.70-1.32)
108-119	0.65 (0.23-1.79)	1.55 (1.43-1.68)	1.21 (0.79-1.85)	0.79 (0.54-1.18)

Multivariable logistic regression used for each outcome as dependent variable and various interpregnancy intervals as independent variables, using the interval 12-23 months as reference. Cases used for analysis: Maternal death model 698960, missing cases 21.9%; preeclampsia model 725718, missing cases 18.9%; eclampsia model 725327, missing cases 18.9%; puerperal Infection model 716624, missing cases 19.9%.

[a]Covariates in final maternal death model: Maternal age, parity (coded 0, 1-3, ≥4), preeclampsia, eclampsia, hemorrhage in 3rd trimester, and previous early neonatal mortality (coded 0, ≥1).

[b]Covariates in final preeclampsia model: Maternal age, parity, diabetes, urinary infection, and previous early neonatal mortality.

[c]Covariates in final eclampsia model: Maternal age, parity, diabetes, urinary infection, previous early neonatal mortality, and previous caesarean.

[d]Covariates in final puerperal infection model: Maternal age, parity, diabetes, preeclampsia, urinary infection, hemorrhage in 1st, 2nd, 3rd trimester and previous early neonatal mortality, and previous caesarean.

Table 1B

Adjusted odd ratios (95% confidence interval) for different perinatal outcomes according to interpregnancy interval in a cohort of 894,476 women delivering two consecutive infants 1990-2009.

Interval (months)	Fetal Death	Neonatal Death	Low Birth Weight	Preterm birth
3-11	1.01 (0.96-1.06)	1.19 (1.09-1.30)	1.07 (1.04-1.10)	1.17 (1.14-1.19)
12-23	Ref	Ref	Ref	Ref
24-35	1.00 (0.94-1.05)	0.99 (0.89-1.08)	1.00 (0.98-1.03)	0.96 (0.94-0.99)
36-47	0.97 (0.91-1.03)	1.03 (0.92-1.14)	0.95 (0.92-0.97)	0.92 (0.90-0.95)
48-59	0.97 (0.91-1.04)	0.87 (0.77-0.99)	0.92 (0.89-0.95)	0.90 (0.88-0.93)
60-71	0.91 (0.85-0.98)	0.93 (0.80-1.07)	0.92 (0.89-0.96)	0.89 (0.86-0.92)
72-83	0.91 (0.84-0.99)	1.00 (0.86-1.18)	0.97 (0.93-1.01)	0.94 (0.91-0.98)
84-95	0.94 (0.86-1.04)	0.84 (0.69-1.02)	1.00 (0.95-1.05)	0.99 (0.94-1.03)
96-107	0.99 (0.89-1.10)	1.02 (0.82-1.25)	1.06 (1.00-1.12)	1.05 (1.00-1.11)
108-119	1.14 (1.01-1.28)	1.07 (0.85-1.36)	1.13 (1.07-1.21)	1.11 (1.05-1.18)

Multivariable logistic regression used for each outcome as dependent variable and various interpregnancy intervals as independent variables, using the interval 12-23 months as reference. Cases used for analysis: Fetal death model 715606, missing cases 20.0%; neonatal death model 630032, missing cases 29.6%; low birth weight model 621214, missing cases 30.5%; preterm birth model 696919, missing cases 22.1%. Covariates in all final models: Maternal age, parity (coded 0, 1-3, ≥4), diabetes, preeclampsia, eclampsia, urinary infection, hemorrhage at 1st, 2nd and 3rd trimesters, and previous early neonatal mortality (coded 0, ≥1) and previous caesarean (coded 0, ≥1).
